# Apolipoprotein E (ApoE) 4 exacerbates pattern separation deficits and amyloid pathology in a combined humanized knock‐in Apolipoprotein E, Amyloid, and Tau model of Alzheimer's Disease

**DOI:** 10.1002/alz70855_099794

**Published:** 2025-12-23

**Authors:** Ariel A Batallán Burrowes, Madison R Longmuir, Aya Arrar, Beatriz G Muratori, Suzete M Cerutti, Lisa M Saksida, Timothy J Bussey, Taylor W Schmitz, Marco AM Prado, Vania F Prado

**Affiliations:** ^1^ University of Western Ontario, London, ON, Canada; ^2^ Robarts Research Institute, London, ON, Canada; ^3^ Schulich School of Medicine & Dentistry, London, ON, Canada; ^4^ Universidade Federal de São Paulo, São Paulo, Brazil; ^5^ Schulich School of Medicine and Dentistry, London, ON, Canada; ^6^ Lawson Health Research Institute, London, ON, Canada

## Abstract

**Background:**

Cognitive deficits in Alzheimer's Disease (AD) are attributed to neuronal network disruptions associated with amyloid β and hyperphosphorylated tau aggregates. Pattern separation, the ability to store and differentiate between similar experiences, is linked to hippocampal function. Pattern separation shows increasing impairment with AD progression. Apolipoprotein E (ApoE) 4 is a significant genetic risk factor for AD, associated with earlier onset, more severe pathology, and cognitive deficits. It is unclear how ApoE4 may interact with AD pathology to affect hippocampal function and pattern separation. This study uses humanized mouse models combining ApoE (3 or 4), App (App, App^NL^_,_ App^NL‐F^), and MAPT (tau) to evaluate the interaction of these factors on pattern separation and amyloid pathology.

**Method:**

Pattern separation was assessed using the automated touchscreen location discrimination (LD) task at ages 6, 9, 12, and 16 months in both male and female mice. Hippocampal amyloid pathology was quantified at the same timepoints using biochemical and immunofluorescence.

**Results:**

Whereas AppApoE3 and ApoE4 mice presented no difference in LD performance at 6 months of age, App^NL^ApoE4 and App^NL‐F^ApoE4 mice demonstrated poorer performance than their ApoE3 counterparts at all ages. When comparing App^NL^ and App^NL‐F^ groups, App^NL‐F^ApoE4 mice exhibited the poorest performance in comparison to the other groups at all ages.

App mice showed no signs of amyloid pathology. App^NL^ApoE3 and App^NL^ApoE4 mice showed no significant difference in insoluble Aβ_42_, and no plaques were detected. In contrast, App^NL‐F^ApoE4 mice displayed increased Aβ_42_ concentrations compared to App^NL‐F^ApoE3, and also presented increased plaque pathology up to 16 months of age.

**Conclusion:**

These results suggest even minor changes in amyloid processing (in App^NL^ mice) seem to synergize with ApoE4 as a driving mechanism of pattern separation deficits, detectable as early as 6 months old. This effect seems to be independent of insoluble amyloid accumulation, which was significantly increased in the hippocampus only after 9 months. Ongoing work examines whether soluble amyloid oligomers and ApoE4's direct impact on neurogenesis contribute to the pattern separation deficits. Nonetheless, we observed a reproducible deficit in LD performance in these mice compatible with early pattern separation deficits.

